# Carbon nanomaterial-based membranes in solid-phase extraction

**DOI:** 10.1007/s00604-023-05741-y

**Published:** 2023-04-06

**Authors:** Chiara Dal Bosco, Massimo Giuseppe De Cesaris, Nina Felli, Elena Lucci, Salvatore Fanali, Alessandra Gentili

**Affiliations:** 1 Department of Chemistry, Sapienza University, P.le Aldo Moro 5, 00185 Rome, Italy; 2 Teaching Committee of Ph.D. School in Nanoscience and Advanced Technologies, University of Verona, Strada Le Grazie, 15 37129, Verona, Italy; 3 Hydro-Eco Research Centre, Sapienza University, Rome, Italy

**Keywords:** Solid-phase extraction, Membranes, Carbon nanotubes, Graphene, Buckypaper

## Abstract

Carbon nanomaterials (CNMs) have some excellent properties that make them ideal candidates as sorbents for solid-phase extraction (SPE). However, practical difficulties related to their handling (dispersion in the atmosphere, bundling phenomena, reduced adsorption capability, sorbent loss in cartridge/column format, etc.) have hindered their direct use for conventional SPE modes. Therefore, researchers working in the field of extraction science have looked for new solutions to avoid the above-mentioned problems. One of these is the design of CNM-based membranes. These devices can be of two different types: membranes that are exclusively composed of CNMs (i.e. buckypaper and graphene oxide paper) and polysaccharide membranes containing dispersed CNMs. A membrane can be used either as a filter, operating under flow-through mode, or as a rotating device, operating under the action of magnetic stirring. In both cases, the main advantages arising from the use of membranes are excellent results in terms of transport rates, adsorption capability, high throughput, and ease of employment. This review covers the preparation/synthesis procedures of such membranes and their potential in SPE applications, highlighting benefits and shortcomings in comparison with conventional SPE materials (especially, microparticles carbonaceous sorbents) and devices. Further challenges and expected improvements are addressed too.

**Graphical Abstract:**

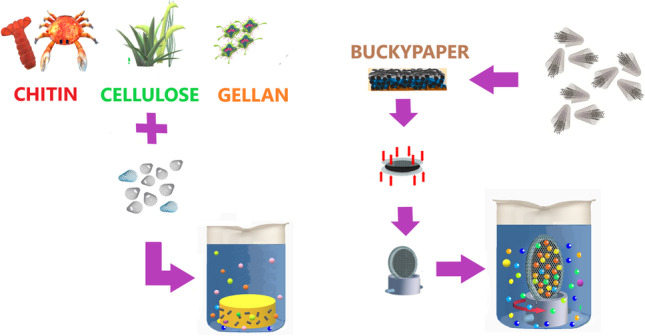

## Introduction

A widely accepted definition encompasses as nanomaterials all materials that have an inner or outer dimension between 1 and 100 nm at least. Thus, nanoobjects include nanoparticles (which are zero-dimensional because all three external dimensions are less than 100 nm), nanofibers/nanotubes (which are mono-dimensional because two outer dimensions are in the nanoscale), and nanoplates (which are bidimensional since only one dimension is in the nanoscale) [1] Based on the previous definition, nanomaterials also comprise nanostructured materials, i.e. materials having external dimensions in the micro- or macro-range but inner dimensions or surface structures in the nanoscale range [2]; examples are nanocomposites, which are composed of polymers entrapping nanoobjects in the bulk [3]; nanosponges, which are porous materials with their internal cavities, pores, or voids, in the nanometre range [4]; agglomerates of nanoobjects, such as buckypaper (BP), composed of carbon nanotube (CNT) bundles [5], and graphene oxide (GO) paper [6].

In the last two decades, both nanoobjects and nanostructured materials have found an increasing use as sorbents for solid-phase extraction (SPE) thanks to their unique properties [1, 7, 8]. Besides low density, their major qualities as sorbents are the large specific surface area, high chemical activity, chemical stability, and easy surface functionalization. Among the different types of nanomaterials that fit the purpose [1, 9] carbon nanomaterials (CNMs) have found wide application, both used individually [10, 11] and in combination with other materials [12–15]. Raw CNMs display a strong affinity towards nonpolar and moderately polar organic compounds, especially when bearing aromatic moieties. A telling example is that of CNTs, which are the most used CNMs in SPE applications. As it is well-known, CNTs look like one graphene (G) sheet (single-walled CNTs, SWCNTs) or multiple G sheets (multi-walled CNTs, MWCNTs) rolled up into cylinders with a hexagonal honeycomb sp^2^ carbon structure. With their nanometre-sized diameters and micrometre-sized lengths, SWCNTs have a length-to-diameter ratio of about 1000, exhibiting a large surface area and high absorption capacity. As a consequence of the distortion of the planar G sheet into a cylinder, the *π*-orbitals of a CNT rearrange in a way that the outer part is much larger than the inner one. Thus, CNTs can exert van der Waals interactions stronger than usual towards organic compounds and effective *π*-*π* interactions with aromatic compounds. On the other hand, for the same reason, they tend to aggregate into bundles because of the extraordinary intertube forces. Each bundle is typically composed of 100–500 tubes and exhibits a lower surface area to volume ratio [16], which trims down their adsorption capability significantly. Unfortunately, this agglomeration phenomenon easily happens packing CNTs into a SPE cartridge: the stationary phase becomes gradually more compacted, increasing backpressure and slowing down the SPE operations (sample loading and analyte elution). Moreover, CNMs (CNTs, G sheets, etc.) may escape from the SPE cartridge, especially under high pressure in online SPE systems. To surmount such limitations, CNTs have been combined with magnetic nanoparticles [17], used to prepare disks [18–20], or supported on/dispersed in membranes [20]. The last ones are very promising solutions, used as disks packed in a cartridge or as a rotating device. Membranes have distinct advantages due to their porosity, high internal surface area, high loading of dispersed CNMs, high transport rates, easy accessibility to active sites, and operational flexibility [21]. In fact, regarding this last aspect, membranes to perform disk-SPE can operate either under the flow-through mode, acting as a filter, or in diffusive mode, acting as a rotating device. The development of such extraction units is still in its infancy, but the literature is already presenting notable examples of CNM-based membranes for SPE applications [22].

This review aims to highlight the latest progress in the design and development of novel SPE devices, based on membranes composed exclusively of CNMs or prepared by dispersing CNMs in a polymeric matrix. Synthesis methodologies of such sorbents as well as their ability in the effective isolation of target compounds from environmental and biological samples are discussed in detail. The evolution and future perspective of such devices are also evaluated.

## Membranes exclusively composed of CNMs

The study of free-standing paper-like materials, based on nanoscale components such as CNTs and GO, has led to the development of BP [23] and GO paper [6]. These relatively new materials (their first preparation dates back to 1998 and 1999, respectively) exhibit macroscopic flexibility and stiffness as a result of a unique interlocking-tile arrangement of their nanoscale components. They have been prepared and applied in several technological sectors as electromechanical actuators [24], biocompatible membranes [25], bioelectrodes [26], and water filtration systems [27]. Only very recently, their utilization has been extended to analytical chemistry as sorbent materials in SPE applications. Table 1 lists some selected applications of membranes exclusively composed of CNMs.

**Table 1:** Selected SPE applications involving the use of carbon nanomaterial-based membranes

Sorbent material	Analytes	Matrix	Extraction procedure	Instrumental technique	Recovery, LOD, LOQ, EF	Ref
Double and triple-layered SWCNTS supported on a qualitative filter paper	Phenols, phthalates, chlorophenols	Tap water, river water, wastewater samples	Disk SPE under flow-through mode	HPLC-FLD and HPLC-DAD	Recovery: 59–100 % LOD: 7–38 ng/L EF = 570–1000	[18]
Double and triple-layered SWCNTs supported on a qualitative filter paper	Sulfonylurea herbicides	Tap water and river water samples	Disk SPE under flow-through mode	HPLC-DAD	Recovery: 76–102% LOD: < 8 ng/L EF = 760–1020	[19]
BP membranes	Humic acids	River water	Disk SPE under flow-through mode	UV/VIS spectrophotometer	Removal efficiency > 93% EF = /	[27]
BP-OASIS HLB	Cobalamins	Cow’s milk	Conventional SPE on cartridge	HPLC/MS/MS	Recovery: 44–100% LOD: 0.36–1.62 µg/L LLOQ: 0.61–2.69 µg/L EF = 17.6–40	[28]
BP membrane	Pesticides and pharmaceutical drugs	River and lake waters	Stir-disk-SPE	HPLC/MS/MS	Recovery: 1–100% LOD: 0.1–12,000 ng/L LOQ: 0.7–40,000 ng/L EF = 1000–100,000	[29]
BP membrane	F2-isoprostanes	Cord and maternal plasma samples	Rotating-disk-SPE	HPLC/MS/MS	Recovery: 30–120% LOD: 1.47–4.06 µg/L LLOQ: 2.45–6.77 µg/L EF = 3–12	[30]
BP membrane	Pesticides, drugs, recreational drugs, and flame retardants	River sediments	Stir-disk-SPE	UHPLC/MS/MS	Recovery: 41–109% LOD: 0.02–3.1 ng/g LOQ: 0.1–9.9 ng/g EF = 81–218	[31]

### SPE modes by using buckypaper

Unlike membranes with vertically aligned CNTs [20, 32, 33], membranes containing bundles of CNTs, such as BP, do not exhibit filtration capability based on size exclusion or sieving in the inner core of the tubes, but rather on the sorption capabilities of the material. The first application of such membranes for extraction purposes goes back to 2008 [18]. In this case, it is not possible to talk about BP in the strict sense of the word because a qualitative filter paper (QFP) was employed as a support to reinforce the obtained sorbent layer and protect it against breaking, which is a weak point of BP and similar material. In order to prepare a disk with a diameter of 47 mm (the common size for a conventional disk for SPE), only 30 mg of SWCNTs was employed. For the analytical application, two stacked disks were used in dynamic flow-through mode to extract phenols, phthalate esters, and chlorophenols from large volumes of environmental waters, without revealing breakthrough problems and obtaining the same extraction capability (90–100%) as a SPE cartridge packed with 500 mg of free MWCNTs [34]. When compared with conventional sorbents (C_18_ and activated carbon disks (500 mg)), the two SWCNT disks could extract chlorophenols more efficiently than the C_18_ disk at a higher sample loading flow rate and required a lower volume of organic solvents than activated carbon disks to desorb the analytes. The same authors prepared a triple-layered SWCNT system to extract sulfonylurea herbicides and other pesticides from water samples [19] after acidification at pH 3.0, with recoveries greater than 76%.

The first actual applications of BP are more recent and were published between 2016 and 2020 [28–31, 34]. The BP used in these investigations is a commercial material, sold as a free-standing paper, composed of bundles of MWCNTs, unoriented and partially oxidized. The paper is a 3D porous network that has a thickness of about two hundred microns. According to the provider information, its preparation is preceded by a step of CNT purification with hydrochloric acid to remove catalyst iron particles, and with nitric acid to wash off amorphous carbons and to oxide CNTs partially. After washing with water, CNTs are suspended in deionized water with the surfactant Triton X-100 and their suspension is pressurized through a filter to form a uniform CNT layer. Compared with the lab-made disks previously described, this method of preparation guarantees membrane uniformity and greater strength avoiding the combination with a support disk. The properties of such commercial BP have been specified in Table 2.

**Table 2:** Main characteristics of buckypaper

Properties of buckypaper	Technique	Ref
A surface area of 110 ± 2 m^2^/g	Brunauer-Emmett-Teller (BET) surface area analysis	[24]
High porosity: 80% with mean size of pores 140 nm. In detail, macropores and mesopores are present, the last ones having a distribution that ranges from 35 to 12.5 Å	Atomic force microscopy (AFM)	[24]
Wettability: BP rapidly absorbs amounts of water up to 4 times its dry weight	Thermogravimetry (TG)	[34]
A permeability of (3.9 ± 0.3)10^−13^ m^2^	(see the reference for the measurement methodology)	[24]
An oxygen/carbon atomic ratio of 0.23 (level of CNT oxidation)	X-ray photoelectron spectroscopy (XPS)	[24]

**Fig. 1:**
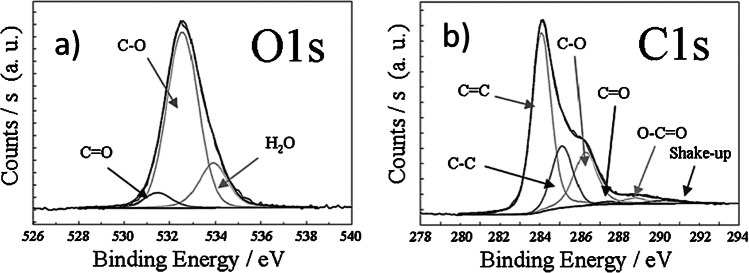
**a** C1s XP spectrum. **b** O1s XP spectrum of BP. The three peaks centred at 286, 287, and 288 eV can be assigned to different types of oxidized carbon: carbon involved in a single bond with an oxygen atom (phenols and ethers); carbon involved in a double bond with an oxygen atom (ketones and aldehydes); carbon involved simultaneously in single and double bond with an oxygen atom (carboxylic groups). (Reproduced with permission from [25])

BP has a specific surface area of about 110 m^2^/g, which is similar to that of microparticle carbonaceous sorbents used in SPE such as graphitized carbon black (GCB) and porous graphitic carbon (PGC) [35]. Unlike PGC, both BP and GCB bear oxygenated groups on their surface which make them able to establish electrostatic interactions and act as ion exchangers; nevertheless, due to the different methods of preparation and oxygen content, their ion exchange capabilities differ [34, 35]: GCB has a low oxygen content, ascribable to γ-pyrone like structures which show basic properties and act as anion exchange sites after activation with hydrochloric acid [29–31, 33, 36–40]; BP has a high oxygen content, mainly represented by carboxylic groups which confer it acid properties and cation exchange capabilities [28]. Moreover, compared with GBC and PGC, BP has a superior sorptive mass capacity due to the larger surface-to-volume ratio. As reported in Table 2, BP is also characterized by high porosity referable to the presence of macropores and mesopores, the last ones spanning from 35 to 12.5 Å, as determined by means of atomic force microscopy [24]; the occurrence of macropores favours the absorption phenomenon, prodromal to the analyte adsorption [28]. Besides, BP has good wettability, a characteristic that is advantageous to preserve the analyte retention even if the sorbent dries out [24, 32]. The high permeability of BP allows the direct treatment of samples from very complex matrices without clogging problems, which is a limitation of the classic SPE on a cartridge. After a 2-h acid treatment with HNO_3_, X-ray photoelectron spectroscopy (XPS) measurements revealed an O/C atomic ratio of 0.23 (see Fig. 1 a and b) [24].

Strictly speaking, BP was used for the first time in combination with a microparticle copolymeric sorbent for the SPE clean-up of cobalamin from milk [28]. To this end, two BP 12-mm disks were packed into the cartridge, separated from the underlying bed of OASIS-HLB by a Teflon frit. The large cross-sectional area and the thin thickness of the disks allow high flow rates without channelling effects. When extractions were performed from Milli-Q water, spiked with the analytes, the yields were very high (82–93%) and more than additive considering that, when the two sorbents were tested individually, BP (13 mg) and OASIS-HLB (500 mg) provided an averaged recovery of 29% and 53%, respectively. Applying the device to the extraction of real samples, after a preliminary step of isoelectric precipitation of caseins at pH 4.6, the relative recoveries, calculated towards an internal standard (dicyanocobinamide) were around 60% that, in the case of problematic analytes such as water-soluble vitamins and a complex matrix such as milk, is a good result. In this work, for the first time, this approach has permitted the characterization of the natural distribution of the vitamin B_12_ homologues in cow’s milk, finding that methylcobalamin is the prevalent form.

With the SPE configuration just considered [28], the dynamic flow-through mode does not permit BP to achieve an adequate contact time with analytes either in the loading or in the elution step, so its adsorption capability is not completely exploited. Thus, to increase the time useful for the analyte adsorption, a completely different configuration has been conceived by preparing different disk-SPE devices, useful to treat environmental samples or biological samples [29–31].

**Fig. 2:**
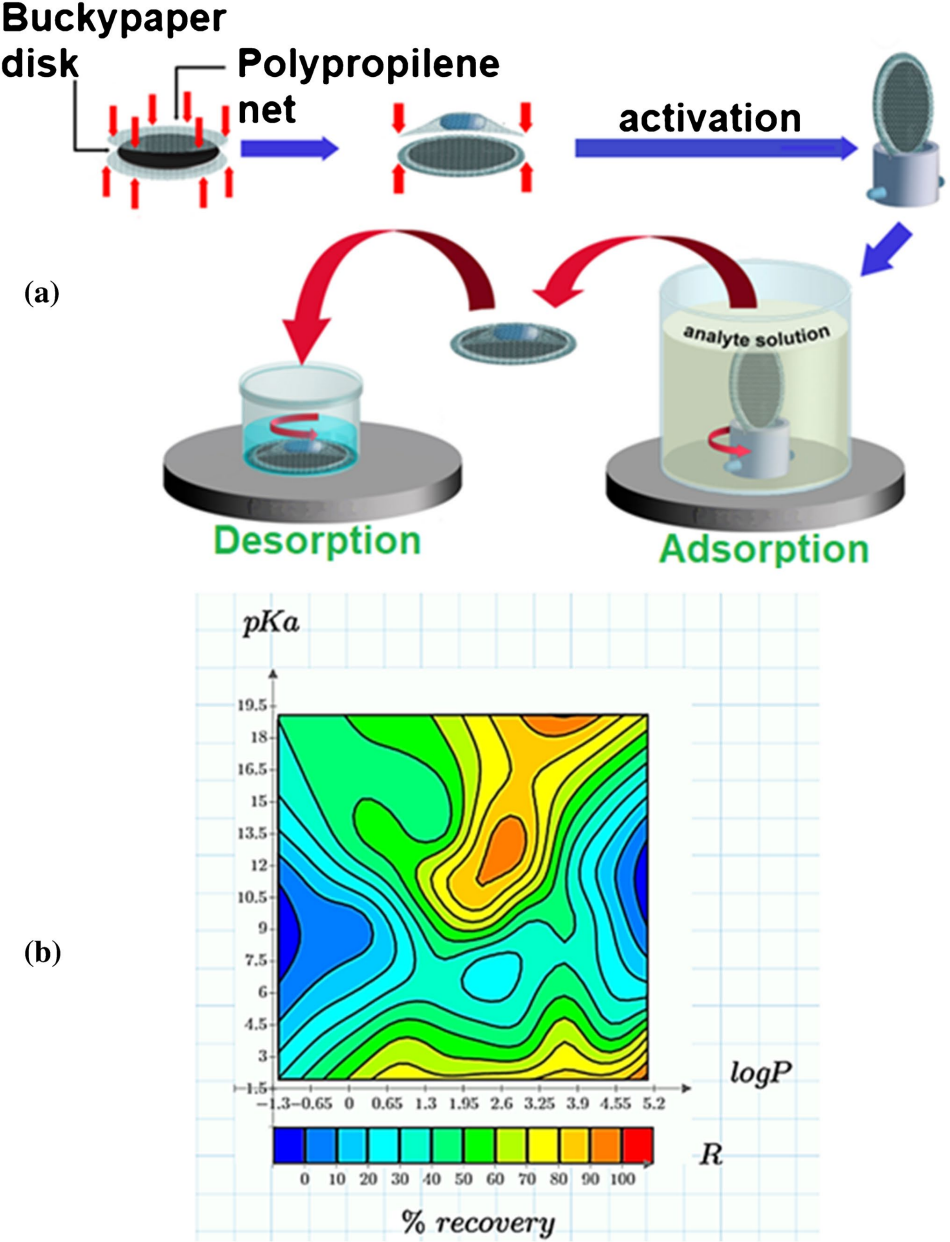
**a** Schematic illustration of the stir-disk SPE unit, components for its assembly, and operational steps. **b** Investigation of the individual influence of logP and pKa on the recovery of the model compounds extracted by the stir-disk SPE device. (Reproduced with permission from [29])

The system shown in Fig. 2a is very effective for processing high sample volumes, such as the case of environmental waters [29]. The first advantage of a such device is that the BP membrane has both sides available for the analyte adsorption and so a diameter of 34 mm is enough to obtain a disk with the same geometric surface as a conventional 47-mm disk that works in flow-through mode using only one side. However, to increase the mechanical integrity and allow the magnetic stirring, the BP disk is inserted into a polypropylene mesh pouch. Before using the device, a step of washing, activation (oxidation), and conditioning is performed as for the conventional SPE on a cartridge involving carbonaceous sorbents, for example GCB. Based on kinetic study outcomes, it has resulted that the adsorption step is controlled by the analyte diffusion into the mesopores of BP. This explains why, unlike the conventional SPE on a cartridge, the adsorption step needs more time to reach analogous recoveries. However, considering the advantage to perform simultaneous extractions on a multi-position stirrer without the strict control of an analyst, a convenient compromise is to realize the adsorption overnight. A shorter desorption time (30 min) is enough for the analyte elution, using a dicloromethane:methanol (50:50, v/v) solution, which is necessary to overcome the strong analyte-BP interactions but it is not an ideal choice from the perspective of green analytical chemistry. Investigating the recovery dependence on the physicochemical properties of different classes of contaminants, it has been observed that logP and pKa are two key parameters [29]. Figure 2 b depicts the level curves correlating absolute recoveries with these parameters and shows that compounds having logP greater than 1 have recoveries between 50% and 100% depending on their pKa. In particular, compounds with pKa greater than 9 and logP between 2 to 4 have a greater probability of being adsorbed on BP, probably due to hydrophobic interaction with its graphenic portion; supplementary interactions such as hydrogen bonds and electrostatic interactions with the polar surface groups of the oxidized BP can improve the adsorption.

**Fig. 3:**
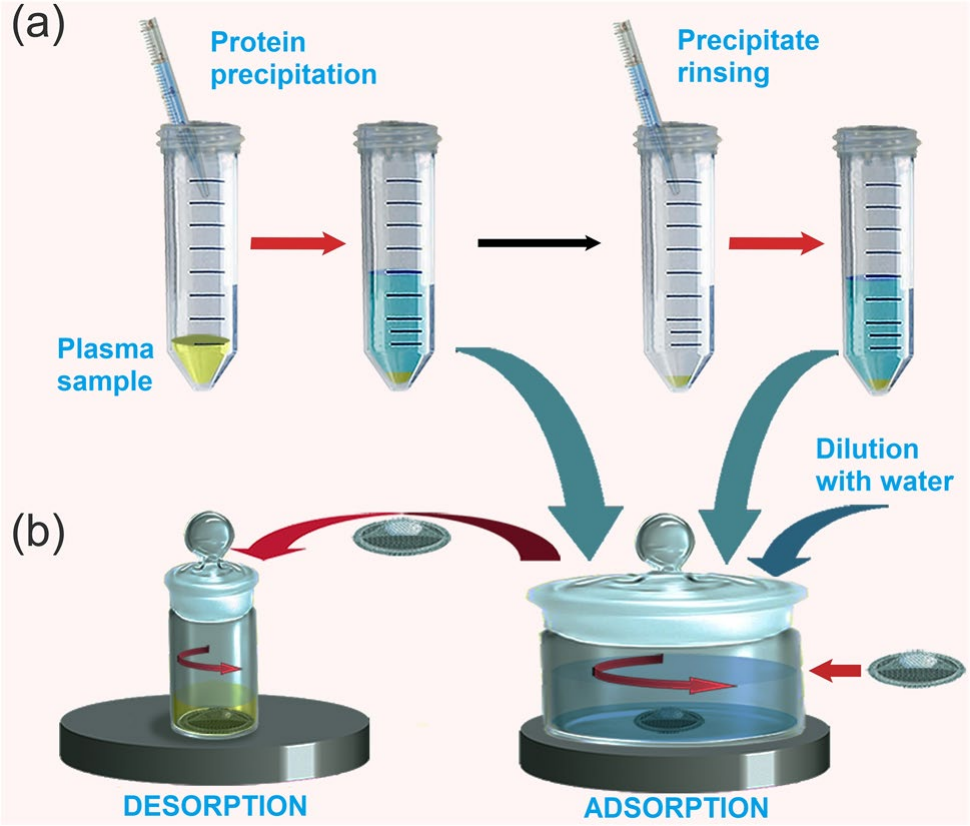
Schematic illustration of the whole extraction procedure: **a** protein precipitation and **b** operational steps of rotating-disk micro-SPE clean-up. (Reproduced with permission from [30])

Further studies have highlighted that the oxidation grade of BP is crucial in affecting extraction yields, especially for those analytes interacting with the polar groups on its surface [30]. As a case study, it was considered the extraction of F2-isoprostanes (log P~4; pKa~4,3) from plasma samples [30]. Figure 3 illustrates the device assembled to perform rotating-disk SPE experiments, suitable for biological applications. Studying the average recovery (R%) of the analytes *vs* activation times (i.e. repeated 2h-cycles of oxidation using nitric acid at 65%), it was observed that a plateau was reached after 6 h of activation with absolute recoveries greater than 80%. This increase in recovery (about 60% higher than that after 2 h of treatment) is explained considering the introduction of hydroxyl, carbonyl, and carboxyl groups on the BP surface. However, after 10 h, the BP disks become fragile because of the prolonged oxidation which causes their rupture [39].

The results obtained in this study indicate the possibility of modulating the extraction capability of BP, but also the possibility of reusing it. It was verified that the recycle is possible eight times. However, since each oxidation treatment modifies the BP surface, a disk can be reused to extract substances with increased polarity.

### The potential of GO paper

As far as the GO paper [6] is concerned, such material has become commercially available only lately, marketed as a circular, flexible black sheet having 4 cm in diameter and 10 µm in thickness. Recently, micrometre-thick films of GO paper have also been named GO membranes. However, despite their great potential in sample preparation, no applications have been developed so far, probably also because of the high price of each disk.

## Polysaccharide-CNM composite membranes

Polysaccharides are a class of biopolymers, characterized by renewable nature, which are biocompatible, biodegradable, available at low-cost, and easy to functionalize; they can also be shaped into different forms, such as films and gels, with a different grade of porosity depending on the preparation technique and % of crosslinking [40]. Polysaccharides easily form gels, i.e. two-component systems in which a liquid is dispersed in a solid. Aerogel, xerogel, hydrogel, and cryogel are gels that can be classified based on the process used to remove the liquid from the solid network of the gel [41]. Since there is often an inappropriate use of the names, Table 3 reports the main types of gels and the corresponding method of preparation.

**Table 3:** Gel classification based on the preparation procedure [41]

Types of gel	Preparation technique
Xerogel	Xerogels are formed following the slow evaporation at room temperature of the liquid of the gel.
Aerogel	Aerogels are formed following the removal of the liquid from gel in its supercritical state.
Cryogel	Cryogels are formed following the removal of the liquid of the gel for sublimation (under vacuum, after freezing of the material).
Hydrogel and alcogel	The terms hydrogel and alcogel are used when the liquid (solvent) in the gel network is water and alcohol, respectively.

Among polysaccharides, those that have been most used to prepare composite membranes/gels with CNMs (polysaccharide matrix@CNM) are chitosan (CS), cellulose, and gellan. Their combination with CNMs is useful not only to obtain a material with an excellent adsorption capability (due to the dispersion of CNMs within the polysaccharide matrix that avoids their aggregation) but also to strengthen the mechanical stability of the resulting composite membrane. Despite the structural similarities between polysaccharides, there are significant differences that allow a considerable grade of flexibility in the membrane preparation. The main of them are related to the polymerization degree, position and/or stereochemistry of the glycosidic bond, occurrence or not of branching, and eventually but of utmost importance, the functional group present at C2 in each saccharide unit (i.e. OH group in cellulose, NH_2_ in CS, etc.).

### CS-based membranes

CS is a linear copolymer obtained by deacetylation of chitin, a highly ordered polysaccharide composing the exoskeletons of insects and crustaceous and the walls of fungi; it is so extremely widespread that 10 Gtons of chitin are constantly distributed in nature [42]. Whereas chitin is made of N-acetyl-D-glucosamine residues linked by β-1,4 linkages, CS is harder to define in terms of its exact chemical composition since it depends on the deacetylation degree. The presence of 2-amino-2-deoxyglucose units makes it soluble in acid solutions through salt formation since the primary aliphatic amine is easily protonated by common organic acids. This cationic polymer can be crosslinked with different reagents taking advantage of the amino group reactivity: anionic crosslinkers (sodium tripolyphosphate, sodium citrate, sulphosuccinic acid, oxalic acid) promote physical interactions, while chemical crosslinkers (genipin, epichlorohydrin, glutaraldehyde) favour covalent bonds. The formation of polymeric networks is a way to avoid its dissolution and to enhance its mechanical properties. Due to its biodegradability, it has also been the most used polysaccharide for extraction purposes over the last years.

**Fig. 4:**
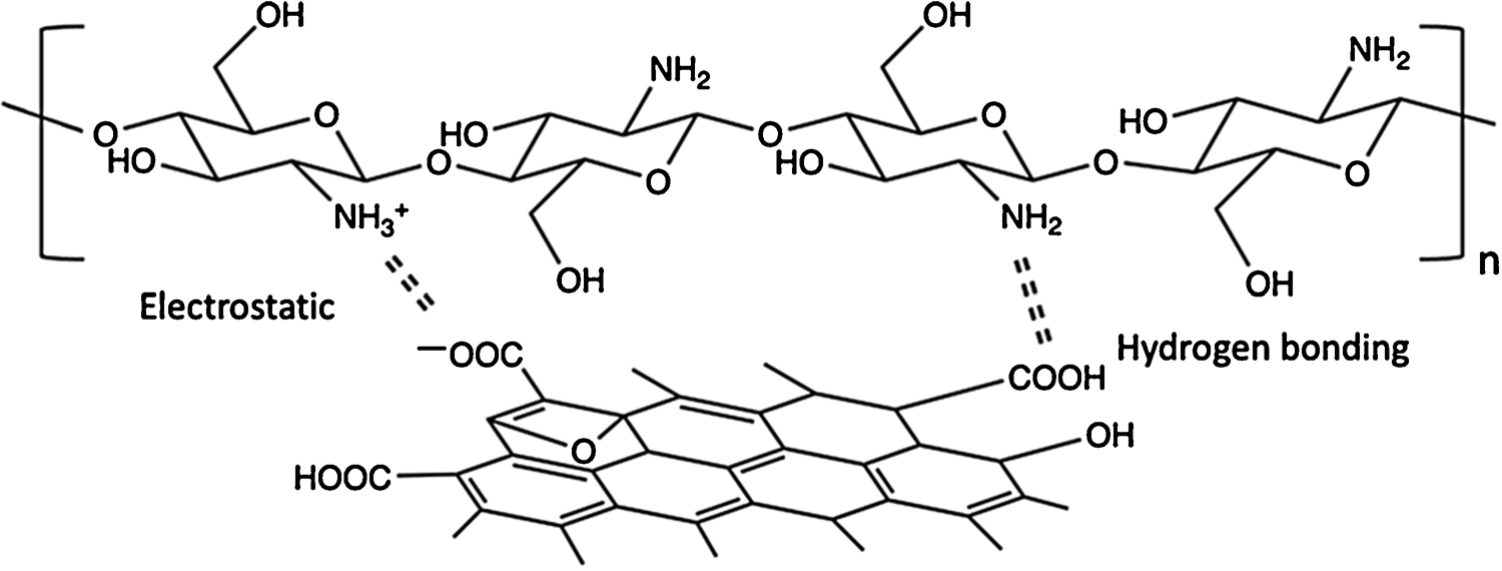
Illustration of interactions between GO and CS (reproduced with permission from [43])

GO/CS composites can be prepared using several strategies, including solvothermal reactions, hydrothermal reactions, and freeze-drying. The formation of hydrogels is favoured by electrostatic interactions between protonated amino groups of CS and the -COO^−^ on GO (at neutral pH), hydrogen bonding, and covalent interactions between CS and GO (see Fig. 4) [43, 44]. Porous CS/GO aerogels prepared by crosslinking and freeze-drying allow one to obtain a material with high porosity (~ 98%), low density (0.021–0.035 g/cm^3^) and high adsorption capacity (about 600 mg/g using methyl orange and amido black 10B as target analytes) [45]. Crosslinking agents, besides improving the mechanical properties of GO/CS, can play a role in the adsorption of pollutants. For example, freeze-dried GO/CS sorbents are more efficient in the retention of dyes when cross-linking is performed after the freeze-drying step due to a higher degree of pore interconnectivity [46].

The solution casting technique was applied to prepare GO/CS composite membranes with different percentages of GO (from 1 to 20% w/w) [47]. Such a technique is a process in which a polymer phase is dissolved in a solvent and mixed with a nanosized material prior to casting on a flat surface (for example a Petri dish). Polymer concentrations of 15–20 w% generally lead to a membrane with a porous structure. In this case, a 2% w/v concentration of CS in 1% v/v acid acetic solution was prepared under stirring and the solvent phase was removed by evaporation after the crosslinking reaction, performed by covering the sample surface with a 0.1% (w/v) glutaraldehyde solution. The resulting crosslinked network exhibited great stability in water. SEM analyses revealed the formation of more homogeneous composite membranes at low GO percentages. However, an improvement in mechanical properties and adsorption capability was observed at high GO contents. The adsorption capability was assessed through recovery experiments of pesticides having different logP (from 0.80 to 6.37) from water samples. Unlike BP, the CS/GO membranes are mechanically more resistant but much less porous and their insertion into a polypropylene pouch is necessary only to fix a magnetic bar in a task on one side of the pouch. Absolute recoveries (R%) using these devices as rotating disks depended on the hydrophobicity of the membranes. The best yields (up to 90%) were achieved by using the membranes containing 20% w/w GO due to the formation of hydrogen bonds and hydrophobic interactions between the analytes and the membrane.

A simple solvothermal synthetic strategy was studied and applied to prepare CS-reduced GO composites with 3D structures (3D CS-rGO) [48]. Solvothermal synthesis is a widely used one-step approach to prepare a wide range of nanostructured materials [49]. In this process, a chemical reaction takes place in a solvent at temperatures above the boiling point and pressures above 1 bar. One of the key features of this method is that temperature and pressure conditions facilitate the dissolution of the chemical reagents and the production of products whose morphology and size can be well controlled. In order to synthesize the 3D CS-rGO (3:17) composite, CS was added to a suspension of GO in ethylene glycol, and then, the mixture was transferred to the solvothermal reactor at 185 °C for 5 h. The solid product was collected by centrifugation, washed, and dried at 70 °C. The resulting sample is an assembly of 2D G sheets into 3D G structures exhibiting superior physical and chemical properties. This is because the 3D network prevents the restacking and aggregation among individual G sheets, guarantees better mass transport, and enhances adsorption performance. The sorbent was applied to extract 70 pesticides from tea samples. Compared with conventional microparticle sorbents such as GCB, C_18_ and primary secondary amine (PSA), 3D CS-rGO allowed the best matrix interference removal and absolute recoveries between 70 and 120%. When applied to analyse catechins from tea, the recovery was 10 times higher than that of GO used alone.

**Fig. 5:**
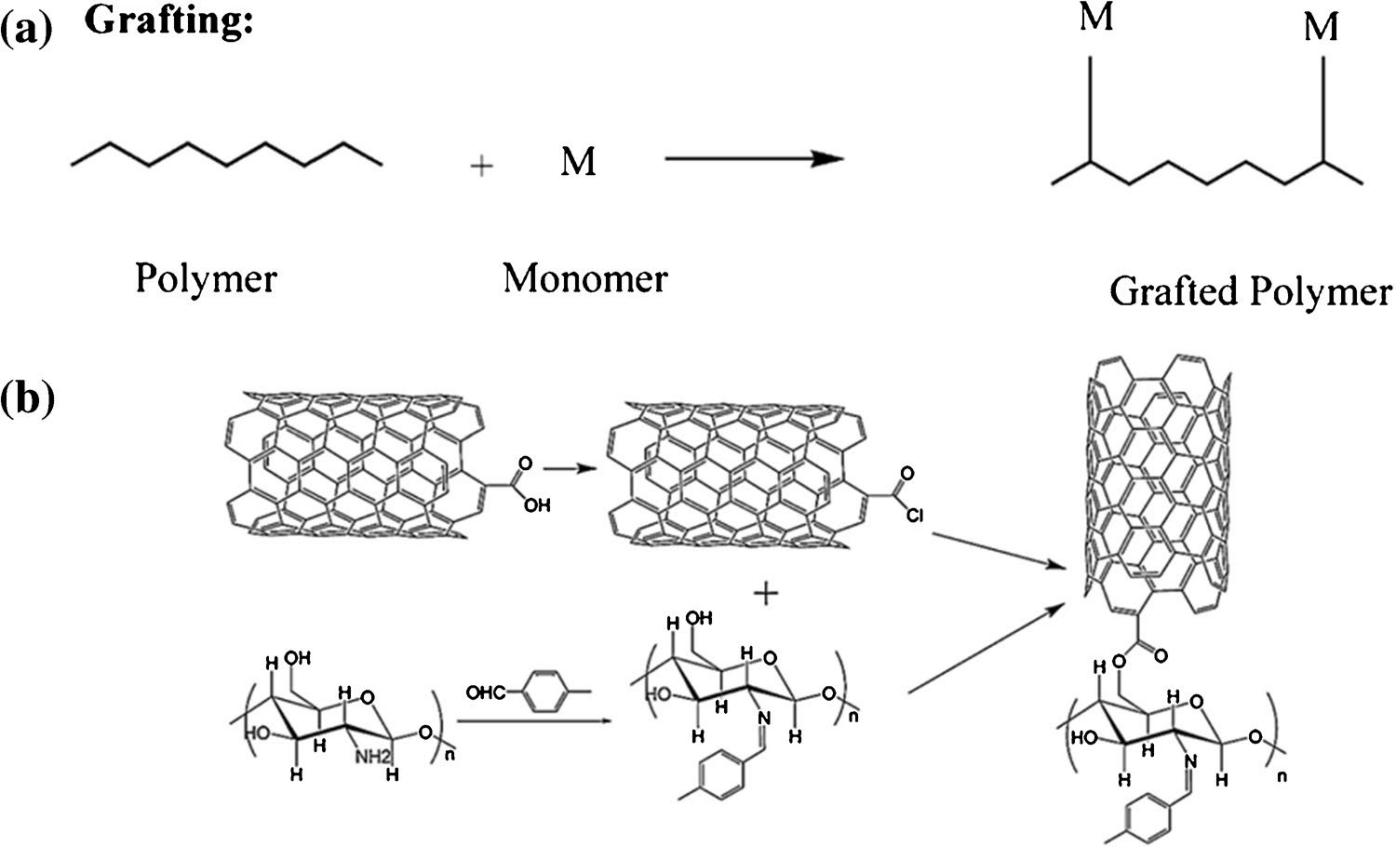
**a** Schematic representation of the grafting method for polymer modification (modified from [51]). **b** Synthesis and structure of S-CS-MWCNTs. (Modified from [50])

CS-based sorbents have also been prepared and applied for the adsorption of metal ions, but not in form of membranes. In a case [50], a SPE column for the extraction and preconcentration of heavy metals from biological and environmental samples was packed with a novel Schiff base-CS-grafted MWCNTs (S-CS-MWCNTs). The grafting process had the aim of improving the chemical stability of CS in acid media and its metal-ion-sorptive properties. Grafting is an effective method to modify (bio)polymers by imparting a variety of functional groups to them; basically, it is a method wherein monomers are covalently bonded onto a polymer chain [51]. Panel a of Fig. 5 schematizes the grafting process, while panel **b** shows the main steps to (i) graft CS with aldehydic groups (S-CS), (ii) synthesize acyl-chloride-modified MWCNTs (MWCNTs-COCl), and finally (iii) prepare the composite sorbent S-CS-MWCNTs [50]. The developed method was applied to the ICP-MS determination of the metal ions in food and environmental samples with quantitative recoveries.

In another case, mercapto-grafted GO–magnetic CS (GO–MCS) was synthesized and used as a sorbent for the magnetic SPE extraction of mercury ions from water samples with quantitative recoveries [52]. Magnetic CS was functionalized with 3-mercaptopropyltrimethoxysilane (MPTS), an effective ligand because the sulphur atom is a non-metal with good affinity towards metals on the right of the d-block; on the other hand, the amine groups of magnetic CS reacted with the carboxyl groups of GO, providing a thiol-functionalized magnetic CS/GO sorbent.

**Fig. 6:**
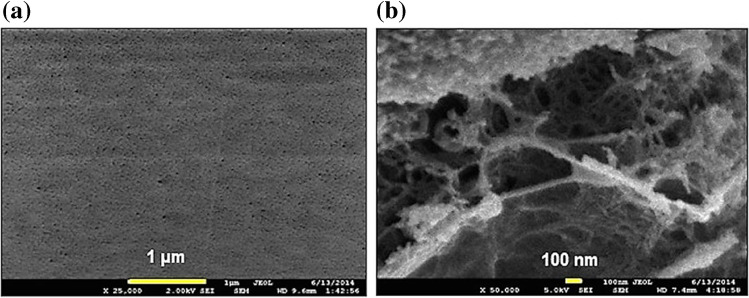
**a** FESEM surface morphology of the AG/CS-MWCNT composite film. **b** the FESEM cross section. (Reproduced with permission from [51])

The possibility of blending different biopolymers simplifies the preparation of a sorbent material with improved chemical properties and mechanical strength. An example is the membrane based on agarose/CS incorporated with MWCNTs (AG/CS-MWCNT) [53]. In this case, chemical properties can be modulated through amino groups of CS and mechanical strength can be enhanced by taking advantage of the high pH resistance of AG. For the membrane preparation, both CS acidified solution and AG solution were individually heated at 90 °C until the complete dissolution of the polymers. After the sequential addition of the aqueous AG solution and MWCNTs to the solution of CS, an aliquot of the warm composite solution was cooled at room temperature in a Petri dish and dried at 60 °C for 48 h. The AG/CS-MWCNT film, characterized by Field Emission Scanning Electron Microscopy (FESEM) (Fig. 6), showed no agglomeration of MWCNTs, which is an advantage in terms of specific surface area, as also confirmed by the BET measurements. This sorbent was applied for the first time for the solid-phase microextraction (SPME) of three non-steroidal anti-inflammatory drugs (NSAIDs) from water samples. In order to perform the SPME procedure, four disks were perforated by a needle which was dipped in an aqueous sample under stirring. The disks were then removed and sonicated with 100 μL of isopropanol, obtaining quantitative recoveries.

### Cellulose-based membranes

Cellulose is the most abundant natural polymer in nature: plants contain on average 33% cellulose with cotton containing the purest form at 90%; other natural sources are also algae, plankton, and bacteria [54]. Cellulose is a linear polymer composed of β-d-glucopyranose units linked by β-(1,4) glycosidic bonds. The most relevant properties of such homopolysaccharide include excellent mechanical properties, superior ability of film and fibre formation, good biocompatibility, and tailorable surface chemistry.

**Fig. 7:**
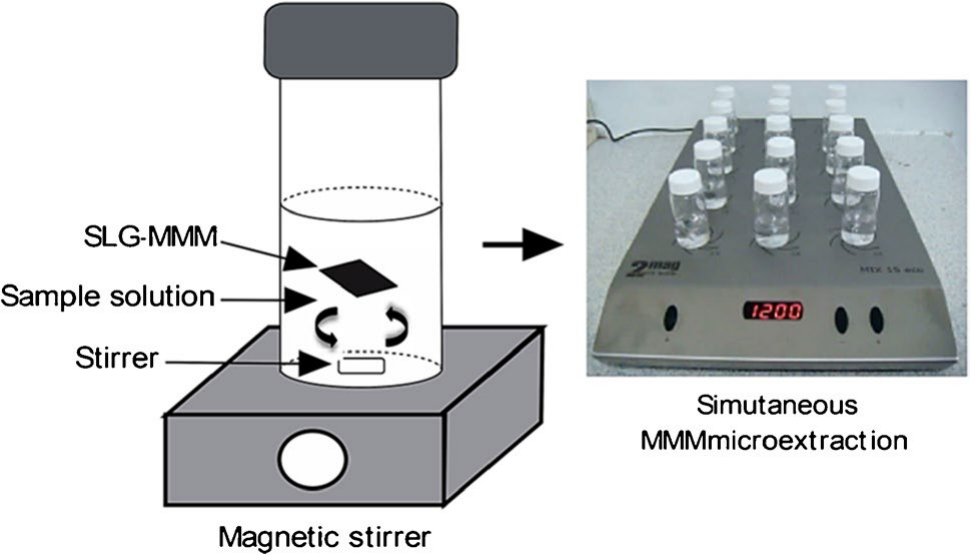
Schematic illustration of a MMM microextraction and the setup for the simultaneous microextraction of 15 samples using a multiposition magnetic stirrer (reproduced with permission from [56])

Several CNTs/cellulose composites were prepared using different strategies and applied for SPE operations: from cellulose-based membranes impregnated with CNTs, which were used to perform the disk-SPE extraction (in flow-through mode) of triazole pesticides from aqueous samples [55], to mixed matrix membranes (MMMs) for the extraction of PAHs from sewage pond water samples [56]. The first ones were prepared simply by percolating a surfactant suspension of CNTs through a cellulose membrane, preliminary oxidized with a hot nitric acid solution. The preparation of MMMs was more articulated. First, MWCNTs and single-layer G were individually dispersed into a cellulose triacetate (CTA) matrix to form a MWCNT-MMM and G-MMM, respectively. Then, the membranes were prepared by casting a solution of the desired proportions of CTA matrix and MWCNTs/G in dichloromethane on a flat glass surface. For recovery studies (see Fig. 7), enrichment factors (EFs) between 54 and 100 were achieved with quantitative recoveries. The performance of such a membrane was also compared with a conventional SPE procedure based on a C_18_ cartridge, obtaining similar values of recoveries, LODs, and LOQs. Finally, the membrane was found to be reusable for a maximum of eight analyses.

### Gellan-based membranes

Gellan, initially known as polysaccharide S-60, is an extracellular bacterial polysaccharide, characterized by a linear anionic tetrasaccharide repeating sequence: two residues of β-d-glucose, one of β-d-glucuronate and one of α-l-rhamnose [57]. The native polysaccharide has an l-glyceryl substituent on O(2) of the 3-linked glucose residue of the tetrasaccharide sequence and, in some repeat units, an acetyl group at O(6) of the same residue. Usually, both substituents are removed for treatment with hot alkali; the resulting deacylated polymer is known generically as “gellan gum” (GG) or Kelcogel (food-grade) or Gelrite (for non-food applications). It is toxic neither to humans nor to the environment. The widespread use of GG is mainly due to its ability to retain water in interstitial spaces among polymer chains. It has been used to prepare composite hydrogels or gel beads as the adsorbents for the sorption studies of dyes [58–61] and heavy metals [62, 63] as well as membranes to catch organic contaminants [60, 64].

Biodegradable GG membranes embedded with MWCNTs were prepared by mixing MWCNTs in a hot GG solution, which was then crosslinked with glutaraldehyde to avoid excessive swelling during the micro-solid-phase extraction (μ-SPE) of PAHs from environmental water and fruit-juice beverage samples [64]. Such membranes allowed an effective good clean-up, quantitative recoveries, and relative standard deviations ≤ 10%. Aerogels composed of GG and GO were synthesized using the freeze-drying approach to obtain 3D structures with different level pore structures depending on the different freezing temperatures: 46% (− 80 °C) and 61% (− 20 °C) [60]. The hierarchical thin sheet structure of the resulting 3D GG@GO aerogels favoured the analyte diffusion, showing excellent performance in methylene blue adsorption. The adsorption process of the dye was spontaneous and exothermic, and it followed pseudo-first-order kinetics and the Freundlich isotherm model. The entire 3D structure was characterized by high thermal stability due to strong electrostatics, hydrogen bonds, and physical entanglement between GG chains and GO.

Table 4 summarizes some relevant SPE applications, based on the use of polysaccharide-CNM composite membranes.

**Table 4:** Selected SPE applications involving polysaccharide-CNM membranes/gels composites

Sorbent material	Analytes	Matrix	Extraction	Instrumental analysis	Recovery, LOD, LOQ, EF	Ref
CS/GO membrane	Pesticides	Water sample	Rotating disk-SP	HPLC/MS/MS	R%:0–90% LOD: / LOQ: / EF = 0–2250	[47]
3D CS-rGO composites	Pesticides	Tea sample	d-SPE	HPLC/MS/MS	R%: 70–120% LOD: 0.01–0.7 µg/L LOQ: 0.04–3.2 µg/L EF = 1.75–3	[48]
S-CS-MWCNTs	Heavy metal ions (V(V), Cr(VI), Cu(II), As(V), and Pb(II))	Herrings, spinach, river, and tap waters	Mini-column SPE	ICP/MS	R%: 91–105% LOD: 1.3–3.8 ng/L LOQ: / EF = 9.1–10.5	[50]
GO-MCS-MPTS	Hg (II)	Tap and seawater samples	d-SPE	FAAS	R%: 95.6–100% LOD: 0.06 µg/L LOQ: 0.12 µg/L EF = 79.7–83.3	[52]
AG/CS-MWCNTs	Non-steroidal anti-inflammatory drugs	Well and river water samples	Dynamic mode via magnetic stirring	HPLC/UV	R%: 94.3–109.7% LOD: 0.89–8.05 ng/mL EF = 94.3–109.7	[53]
Cellulose membrane impregnated with CNTs	Triazole fungicides	Lake water samples	Disk-SPE	GC/MS	R%: / LOD: 0.02–0.03 µg/L LOQ: 0.05–0.1 µg/L EF > 2500	[55]
MWCNT-MMM and SLG-MMM (cellulose triacetate membrane)	PAHs	Sewage pond water sample	Dynamic mode via magnetic stirring	HPLC/UV	R%: 99–100.5% LOD: 0.02–0.09 ng/mL LOQ: 0.06–0.28 ng/mL EF = 123.75–125.6	[56]
MWCNTs-GG	PAHs	Environmental waters and fruit juice beverage sample	Dynamic mode via magnetic stirring	HPLC/UV	R%: 81.7–106.5 % LOD: 0.01–0.06 µg/L LOQ: 0.04–0.2 µg/L EF = 326.8–426	[64]

## CNM-based membranes with enhanced selectivity

In the last years, two opposite tendencies have become apparent within the field of extraction science: from one side, there is the necessity of isolating the maximum possible number of compounds simultaneously to perform multi-class, multi-analyte methods, mainly based on liquid chromatography-mass spectrometry (LC-MS); on the other side, there is the necessity of extracting a series of compounds with similar physicochemical compounds from different complex samples. In the first case, the poor selectivity of CNMs as SPE sorbents is an advantage which allows one to carry out analyses on large scale accepting compromise on recovery yields. In the second case, the use of sorbents, modified or combined with other materials to enhance their selectivity, is an added value to perform targeted analyses. From this point of view, several researchers have been working to develop even more specialized CNM-based sorbents, for example by resorting to the combination of CNMs with metal-organic frameworks (MOFs) [65–67] and molecularly imprinted polymers (MIPs) [68, 69].

MOFs are microporous hybrid materials with infinite framework structures built from organic linkers and inorganic metal nodes. They are characterized by a high surface area and uniform nanostructured cavities. These peculiarities make MOFs suitable SPE sorbents since their pores and cavities can be tailored for the selective adsorption of analytes on the basis of size and/or interaction with the framework [70]. Interesting examples of hybrid sorbent are those composed of MOF-199, a copper-based MOF, combined with various CNMs such as G and fullerene [66]. Such materials were applied for the d-SPE of PAHs from water samples with recoveries greater than 92%. The MOF/G composite showed the greatest adsorption affinity probably due to the high porosity of G. However, there are few examples of hybrid membranes prepared with both these nanomaterials, not yet designed for SPE but rather for water purification [71]. One of these is the UiO-66@GO/polyethersulfone membrane prepared for water ultrafiltration [71]. UiO-66 was specifically anchored to the GO layers as a porous modifier and to prevent the GO layers from stacking. The aperture size of UiO-66 is about 6.0 Å, which falls between the size of water molecules (∼ 2.8 Å) and most organic contaminants (greater than 6.0 Å); as a result, the membrane UiO-66@GO/PES exhibited high hydrophilicity and water purification performance.

Using MIP-based sorbent, the selectivity of SPE is exasperated to the point that MIPs have been compared to synthetic antibodies [68]. The shortcomings of traditional bulk imprinting of MIPs (partial template removal, limited site accessibility, low mass transfer, and binding capacity) have been overcome by surface molecular imprinting [69, 72]. Such a technology involves the occurrence of polymerization reaction on the surface of solid-phase matrixes. During surface polymerization, the imprinted sites are created on the surface of (bio)polymers or solid substrates. The stereo cavities in the imprinted layer act as specific recognition sites and can selectively adsorb target analytes from complex samples [73]. Core–shell structures have been prepared depositing imprinted polymers on a core-shell structure with a large surface area and high porosity, such as CNTs, GO, and carbon spheres, so to obtain surface imprinted composite beads [72]. Multitemplate molecularly imprinted biopolymers were prepared using CS as the biopolymer to be imprinted and carbon nanospheres as the core [69]; the beads were then used as sorbents for the ultrasound-assisted d-SPE of vitamins B2, B3, and B6 from juice samples with recoveries greater than 76%. The surface imprinting technique has also been used to synthesize molecularly imprinted membranes (MIMs) for water purification [72] or as a dispersant sorbent to realize the matrix solid-phase dispersion (MSPD) for extraction of parabens from powder sunscreen samples [74]. Nevertheless, despite the great potentiality of the surface molecular imprinting technique in the preparation of specialized membranes, there are no examples of composites involving CNMs so far, probably because this kind of research is still moving its first steps.

## Conclusion and perspectives

Membranes represent an alternative way to perform SPE and to overcome the limitations of the conventional procedure carried out on a cartridge in flow-through mode. Besides the improvement of the analytical figures of merit arising from smart uses of CNMs, the main practical advantages related to the employment of membranes are the great simplification of the SPE operations and the possibility of processing tens of samples simultaneously resorting to low-cost instrumentation (for instance a multiposition magnetic stirrer) and with a minimal effort of analysts. Safety aspects are also improved because the dispersion of CNMs in the atmosphere is avoided both using BP and polysaccharide matrices. An apparent limitation of CNM-based membranes might be identified in their poor selectivity because nonpolar compounds (particularly aromatic compounds) are retained via *π*-*π* interactions. However, low specificity is a characteristic common also to a large family of microparticle sorbents (silica-based gels such as C_18_, C_8_, etc.; porous polymer sorbents such as poly(styrene-divinylbenzene), poly(divinylbenzene-N-vinylpyrrolidone), etc.; carbonaceous sorbents), which can be seen as a benefit when the objective is to realize multi-class multi-analyte analyses, usually via HPLC/GC-MS. More specific sorbents can be obtained either exploiting the ease with which oxidized CNMs can be derivatized or preparing composite sorbents with marked selectivity through the combination with MIPs and MOFs. Real weak points of CNM-based membranes can be identified in the brittleness of the BP disk borders or in the difficulty in removing water from polysaccharide membranes before the desorption step due to the hydrophilic nature of the matrix.

Other characteristics such as recyclability make these devices worthy of attention. For instance, BP can be reused several times, allowing cost amortization, and making it a competitive product for sample preparation. Due to their ability in forming physical and chemical porous hydrogels, there is growing interest in using polysaccharides to create functional materials from naturally available biomass; after their use, polysaccharide membranes are easily biodegradable. Different considerations can be formulated regarding CNM recyclability which is not a trivial issue. To date, no effective method has been reported for their removal from liquid systems. It is easy to remove most CNMs from an aqueous solution by filtration, but a few types may pass through the filter pores and enter the environment. A sustainable method could be that of recycling CNMs which are already well-dispersed in scrap materials, so that they can be integrated into new ones readily, using less energy and chemicals. Recently, some approaches have been proposed to completely degrade CNTs at the end of their life cycle. For example, the biodegradation of CNTs using horseradish peroxidase and myeloperoxidase through the formation of sodium hypochlorite or hypochlorous [75]. The direct use of these reagents has also been proposed since they are inexpensive and environmentally friendly oxidizing agents able to completely degrade CNTs into carbon oxides or carbonate ions [76].

Although BP and GO paper are commercially available, several types of CNM-based membranes for SPE are still lab-made. The main challenge for the mass production of such membranes is how to integrate CNMs with their unique properties into a robust membrane structure with outstanding separation performance. Other major limitations include CNT dispersion, reproducibility among batches, presence of defects at the polymer/CNT interface and compatibility between the polymer and CNTs. Consequently, the homogeneous bulk fabrication of CNM-based membranes is still at a premature stage and requires further research to improve the technology for their scaled-up production [77–79].

Although the adoption of membranes in SPE is still at the development phase, this approach is very promising, can involve the preparation of more and more sophisticated devices (for instance, self-rotating systems following the incorporation of magnetic nanoparticles), and may find a host of applications in the immediate future.

## Abbreviations

*3D CS-rGO*: chitosan-reduced graphene oxide composites with 3D structures
*4-NP*: 4-n-nonylphenol
*4-OP*: 4-tert-octylphenol
*AFM*: atomic force microscopy
*AG/CS-MWCNT*: composite membrane based on agarose/chitosan incorporated with multiwalled carbon nanotubes
*BET*: Brunauer-Emmett-Teller analysis
*BP*: buckypaper
*BPA*: bisphenol A
*CNMs*: carbon nanomaterials
*CNT*: composed of carbon nanotube
*CS*: chitosan
*CTA*: cellulose triacetate
*EF*: enrichment factors
*FAAS*: flame atomic absorption spectrometry
*FESEM*: field emission scanning electron microscopy
*G*: single-layer graphene
*GCB*: graphitized carbon black
*GC-MS*: gas chromatography-mass spectrometry
*GG*: gellan gum
*GO*: graphene oxide
*GO-MCS-MPTS*: graphene oxide-magnetic chitosan functionalized with 3-mercaptopropyltrimethoxysilane
*HPLC-FLD*: high-pressure liquid chromatography-fluorescence detector
*HPLC-DAD*: high-pressure liquid chromatography-diode array detector
*HPLC/GC-MS*: high-pressure liquid chromatography/gas chromatography-mass spectrometry
*HPLC-MS/MS*: high-pressure liquid chromatography-tandem mass
*HPLC-UV*: high-pressure liquid chromatography with ultraviolet
*ICP-MS*: inductively coupled plasma-mass spectrometry
*LC-MRM*: liquid chromatography-multiple reaction monitoring
*LODs*: limit of detections
*LLOQ*: lower limit of quantifications
*LOQs*: limit of quantifications
*MIMs*: molecularly imprinted membranes
*MISPE*: molecularly imprinted SPE
*MMM*: mixed matrix membrane
*MPTS*: 3-mercaptopropyltrimethoxysilane
*MWCNTs*: multi-walled carbon nanotubes
*MWCNTs-COCl*: acyl-chloride-modified multiwalled carbon nanotubes
*MWCNTs-GG*: composite multiwalled carbon nanotubes-gellan gum
*MMM-MWCNTs*: mixed matrix membrane-multi-walled carbon nanotubes
*NSAIDs*: non-steroidal anti-inflammatory drugs
*PAHs*: polycyclic aromatic hydrocarbons
*PGC*: porous graphitic carbon
*PSA*: primary secondary amine
*QFP*: qualitative filter paper
*S-CS-MWCNTs*: Schiff base-chitosan-grafted multiwalled carbon nanotubes
*SLE*: solid-liquid extraction
*SLG-MMM*: single-layer graphene-mixed matrix membrane
*SDS*: sodium dodecyl sulphate
*SPE*: solid-phase extraction
*SPME*: solid-phase microextraction
*SWCNTs*: single-walled carbon nanotubes
*TG*: thermogravimetry
*UHPLC/MS/MS*: ultra-high-pressure liquid chromatography-tandem mass
*XPS*: X-ray photoelectron spectroscopy
*μ-SPE*: micro-solid-phase extraction
